# Plasma biomarkers of Alzheimer’s disease improve prediction of cognitive decline in cognitively unimpaired elderly populations

**DOI:** 10.1038/s41467-021-23746-0

**Published:** 2021-06-11

**Authors:** Nicholas C. Cullen, Antoine Leuzy, Shorena Janelidze, Sebastian Palmqvist, Anna L. Svenningsson, Erik Stomrud, Jeffrey L. Dage, Niklas Mattsson-Carlgren, Oskar Hansson

**Affiliations:** 1 Clinical Memory Research Unit, Lund University, Lund, Sweden; 2 Memory Clinic, Skåne University Hospital, Lund, Sweden; 3 Eli Lilly and Company, Indianapolis, IN USA; 4 Department of Neurology, Skåne University Hospital, Lund, Sweden; 5 Wallenberg Centre for Molecular Medicine, Lund University, Lund, Sweden

**Keywords:** Alzheimer's disease, Neurodegeneration, Predictive markers

## Abstract

Plasma biomarkers of amyloid, tau, and neurodegeneration (ATN) need to be characterized in cognitively unimpaired (CU) elderly individuals. We therefore tested if plasma measurements of amyloid-β (Aβ)42/40, phospho-tau217 (P-tau217), and neurofilament light (NfL) together predict clinical deterioration in 435 CU individuals followed for an average of 4.8 ± 1.7 years in the BioFINDER study. A combination of all three plasma biomarkers and basic demographics best predicted change in cognition (Pre-Alzheimer’s Clinical Composite; R^2^ = 0.14, 95% CI [0.12–0.17]; P < 0.0001) and subsequent AD dementia (AUC = 0.82, 95% CI [0.77–0.91], P < 0.0001). In a simulated clinical trial, a screening algorithm combining all three plasma biomarkers would reduce the required sample size by 70% (95% CI [54–81]; P < 0.001) with cognition as trial endpoint, and by 63% (95% CI [53–70], P < 0.001) with subsequent AD dementia as trial endpoint. Plasma ATN biomarkers show usefulness in cognitively unimpaired populations and could make large clinical trials more feasible and cost-effective.

## Abstract (web summary)

Plasma biomarkers of amyloid, tau and neurodegeneration (ATN) need to be characterized in cognitively unimpaired (CU) elderly individuals. Here, the authors show plasma ATN biomarkers predict clinical deterioration and cognitive decline and show in a simulated clinical trial combining all three biomarkers reduced the required sample size.

## Introduction

Alzheimer’s disease (AD) is characterized by the presence of amyloid-β (Aβ) plaques and tau tangles^1^. Specifically, Aβ is thought to potentiate the spread of neocortical tau pathology which in turn drives neurodegeneration and cognitive decline^2^. Accruing evidence indicates, however, that these changes are slow and protracted and precede the initial symptoms of AD by many years or decades^3,4^. A prerequisite for the testing of therapeutic interventions is the identification of individuals at risk for progression to AD dementia. Biomarkers have been explored for this^5^. The National Institute on Aging and Alzheimer’s Association has proposed a framework for research wherein cerebrospinal fluid (CSF) and imaging (magnetic resonance and positron emission tomography (PET)) based measures of Aβ (A), tau (T), and neurodegeneration (N) can be compiled into an ATN classification system^6^. CSF measures include the Aβ42/Aβ40 ratio, tau phosphorylated at threonine 181 (P-tau181) and neurofilament light (NfL). Continued assay development now makes it possible to measure ATN biomarkers in blood, including the Aβ42/Aβ40 ratio^7,8^, P-tau^9–11^ and NfL^12,13^. With lower cost and higher accessibility, blood-based AD biomarkers may circumvent the limitations inherent to CSF and imaging. While recent work on plasma biomarkers has primarily focused on individualized risk assessment in patients with mild cognitive impairment (MCI)^14–16^, it has been noted that substantial irreversible neuronal loss is already seen by this stage, which may reduce the likelihood of disease-modifying therapies to prevent dementia onset^17^. This has led to an increasing focus on cognitively unimpaired (CU) older individuals at risk for progression to AD dementia on the basis of biomarker evidence of brain AD pathology^18^. Studies on combinations of blood-based Aβ42/Aβ40, P-tau and NfL have reported findings on their diagnostic accuracy for the separation of AD dementia from CU individuals and patients with non-AD disorders^7,9,11,19–22^, but data are lacking on their performance in predicting cognitive decline and clinical progression in CU individuals. We therefore tested the usefulness of plasma Aβ42/Aβ40, P-tau217, and NfL (separately and combined) for predicting longitudinal cognitive decline and clinical outcomes in a CU population comprised of both cognitively normal (CN) healthy controls and individuals with subjective cognitive decline (SCD) who did not meet the diagnostic criteria for MCI. We hypothesized that a combination of ATN plasma biomarkers could improve the prediction of cognitive decline and risk of AD dementia compared to using only basic demographic information. We also investigated the extent to which plasma biomarkers could reduce the sample size required to adequately run AD clinical trials in a CU elderly population.

## Results

### Study population characteristics

A total of 435 CU individuals were included of which 167 (38.4%) had SCD. During follow-up, a total of 28 individuals converted to AD dementia (6.4%) and 39 individuals converted to all-cause dementia (9.0%). SCD participants had significantly lower baseline Mini-Mental State Examination (MMSE, *P* < 0.001) and Preclinical Alzheimer’s Cognitive Composite (PACC, *P* < 0.001) scores, a significantly higher risk of AD dementia (*P* < 0.001), and significantly higher baseline plasma NfL levels (*P* = 0.04). However, there was no difference in longitudinal change in MMSE (*P* = 0.41) or PACC (*P* = 0.10), nor in baseline plasma Aβ42/Aβ40 (*P* = 0.72) or P-tau217 (*P* = 0.28) levels, between CN and SCD participants. Study population characteristics are provided fully in Table 1.

**Table 1:** Study participant characteristics.

	Overall	CN	SCD	*P* value
*n*	435	268	167	
Age, years	72.58 (5.45)	73.62 (5.01)	70.92 (5.73)	<0.001
Education, years	12.17 (3.66)	12.12 (3.74)	12.27 (3.53)	0.59
Sex = Male (%)	242 (55.6)	159 (59.3)	83 (49.7)	0.095
1+ *APOE* ε4 (%)	147 (34)	73 (27)	74 (45)	0.001
MMSE, baseline	28.80 (1.21)	29.03 (0.93)	28.44 (1.47)	<0.001
MMSE, four-year change	−1.02 (2.93)	−0.65 (1.79)	−1.68 (4.18)	0.414
PACC, baseline	0.01 (0.74)	0.17 (0.70)	−0.23 (0.75)	<0.001
PACC, four-year change	−0.33 (0.85)	−0.27 (0.73)	−0.44 (1.05)	0.102
Follow-up time, years	4.75 (1.66)	4.99 (1.58)	4.35 (1.71)	<0.001
Converted to AD dementia (%)	28 (6.4)	3 (1.1)	25 (15.0)	<0.001
Converted to any dementia (%)	39 (9.0)	6 (2.2)	33 (19.8)	<0.001
Plasma Aβ42/Aβ40, baseline	66.08 (7.28)	66.26 (7.47)	65.77 (6.97)	0.72
Plasma P-tau217, baseline	4.97 (0.81)	5.00 (0.77)	4.92 (0.88)	0.283
Plasma NfL, baseline	7.59 (0.46)	7.63 (0.45)	7.54 (0.47)	0.036
CSF Aβ42/Aβ40, baseline	7.13 (0.50)	7.15 (0.48)	7.09 (0.53)	0.38
CSF P-tau181, baseline	2.96 (0.40)	2.94 (0.36)	3.00 (0.45)	0.179
CSF NfL, baseline	6.79 (0.45)	6.75 (0.42)	6.86 (0.50)	0.027

This table shows the characteristics for the study population. All variables are presented as “mean (standard deviation)” unless otherwise noted as a percentage “(%)” in the table. All biomarkers besides plasma Aβ42/Aβ40 were natural log-transformed prior to analysis and all biomarker values are provided in terms of pg/mL. *P* values represent a comparison between CN and SCD groups: *t*-tests for continuous and chi-square tests for non-continuous measures. All statistical tests were two-sided with no adjustment for multiple comparisons.

*CN* cognitively normal (healthy controls), *SCD* subjective cognitive decline, *Aβ42/Aβ40* the 42- divided by the 40-amino acid long beta-amyloid peptide, *P-tau217 (P-tau181)* the (181-) 217-amino acid long phosphorylated tau peptide, *NfL* neurofilament light chain.

### Association between plasma biomarkers

When testing the associations between unadjusted plasma biomarker values using Spearman correlation (Supplementary Fig. 1), there was a significant correlation between Aβ42/Aβ40 and P-tau217 (*ρ* = −0.22, *P* < 0.0001), between P-tau217 and NfL (*ρ* = 0.20, *P* = 0.0005), and between NfL and Aβ42/Aβ40 (*ρ* = −0.10, *P* = 0.03).

A total of 201 participants (46.2%) were plasma Aβ42/Aβ40-positive, 162 participants (37.2%) were plasma P-tau217-positive, and 165 participants (37.9%) were plasma NfL-positive.

### Modeling longitudinal change in cognition

**Fig. 1:**
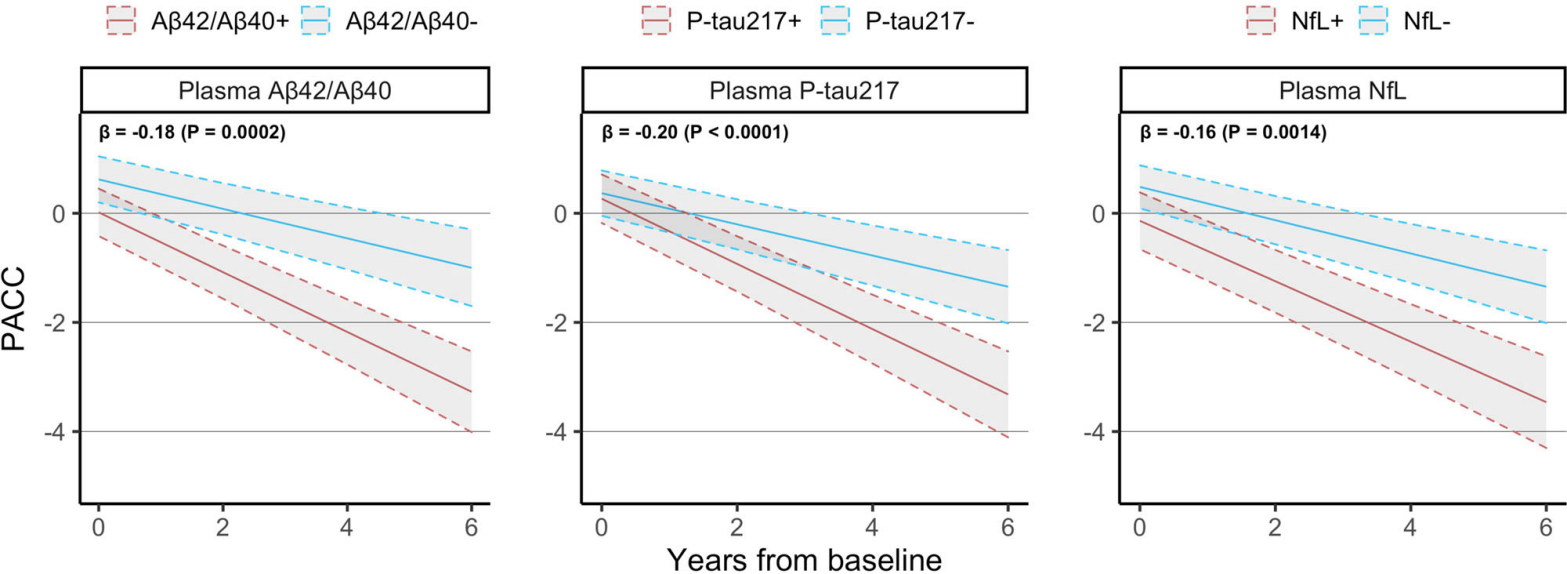
Relationship between plasma biomarkers and change in PACC. This figure shows the longitudinal PACC trajectory expected for a biomarker-negative or biomarker-positive CU individual with average age, average education, and female sex. β-coefficients at the top left of each panel are presented in terms of “points/year per standard deviation change in biomarker value” and are derived from linear mixed effects models with longitudinal PACC as outcome and age, sex, education, plus each plasma biomarker included separately from each other as predictors. All statistical tests were two-sided with no adjustment for multiple comparisons. Shaded areas represent 95% confidence intervals of the regression lines.

We first tested whether plasma biomarkers were significantly associated with cognitive decline as measured by the PACC. When added individually to a basic model consisting of age, sex, and education (Fig. 1), we found that plasma P-tau217 had the strongest association with longitudinal change in PACC (*β* = −0.20 points per year per SD increase in biomarker value, *P* < 0.0001), followed by plasma Aβ42/Aβ40 (β = −0.18, *P* = 0.0002), and plasma NfL (β = −0.16, *P* = 0.001). When combining all three plasma biomarkers together in the same model (Table 2), all three biomarkers remained significant (*P* = 0.004 for Aβ42/Aβ40, *P* = 0.002 for P-tau217, *P* = 0.01 for NfL). Adding *APOE* ε4 status to the basic model did not affect the significance of any biomarkers in either the combined or individual models (Supplementary Table 1), although the effect of plasma Aβ42/Aβ40 in the combined model decreased the most (*β* = −0.16, *P* = 0.002 with *APOE* ε4 status).

**Table 2:** Association between plasma biomarkers and longitudinal PACC.

Model	Beta coefficient			*R*^2^ [95% CI]	Ref: basic model	
	Plasma Aβ42/Aβ40	Plasma P-tau217	Plasma NfL		*P* value	AIC_Δ_
ATN	−0.15 [−0.24, −0.05] (*P* = 0.0026)	−0.15 [−0.25, −0.06] (*P* = 0.0020)	−0.12 [−0.21, −0.02] (*P* = 0.0141)	0.14 [0.12, 0.17]	<0.0001	−28
A	−0.18 [−0.19, −0.09] (*P* = 0.0002)			0.11 [0.09, 0.14]	<0.0001	−14
T		−0.20 [−0.20, −0.11] (*P* < 0.0001)		0.09 [0.08, 0.13]	0.0002	−13
N			−0.16 [−0.25, −0.06] (*P* = 0.0014)	0.10 [0.08, 0.14]	0.002	−9

This table shows the results from fitting linear mixed effects models with longitudinal PACC as outcome and plasma biomarkers added separately or all together to a basic model consisting of age, sex, and education. β-coefficients are presented in terms of “PACC points/year per standard deviation change in biomarker value.” *R*^2^ values were evaluated and confidence intervals were calculated using 1000 bootstrapped samples. The basic model consisting of only demographics had *R*^2^ = 0.07 (95% CI [0.06, 0.11]) and AIC = 6699. *P* values represent an ANOVA comparison to the basic model; AIC_Δ_ values represent the change in AIC compared to the basic model and an AIC_Δ_ value of −2 or lower implies a better fit than the basic model. All statistical tests were two-sided with no adjustment for multiple comparisons.

All plasma biomarker models significantly improved prediction of PACC change compared to the basic model (AIC = 6699; *R*^2^ = 0.07, CI [0.06, 0.11]; see Table 2). In terms of AIC, a combination of all three plasma biomarkers predicted PACC change best (AIC_Δ_ = −28 versus basic model; *R*^2^ = 0.14 [0.12, 0.17], *P* < 0.0001), followed by plasma Aβ42/Aβ40 only (AIC_Δ_ = −14; *R*^2^ = 0.11 [0.09, 0.14], *P* < 0.0001), plasma P-tau217 only (AIC_Δ_ = −13; *R*^2^ = 0.09 [0.08, 0.13], *P* = 0.0002), and finally plasma NfL only (AIC_Δ_ = −9; *R*^2^ = 0.10 [0.08, 0.14], *P* = 0.002).

A sensitivity analysis in which longitudinal MMSE was used as the cognitive outcome showed an increased effect of plasma NfL and a decreased effect of plasma Aβ42/Aβ40, while plasma P-tau217 remained a strong predictor (Supplementary Fig. 2; Supplementary Table 2). All biomarkers remained significant when combined together. Another sensitivity analysis in which we additionally adjusted for the effect of SCD on both baseline and change in cognitive resulted in a greatly increased baseline model fit (*R*^2^ = 0.17), while this did not affect the significance of any plasma biomarkers nor the significant improvement in model fit found by adding plasma biomarkers to the basic model (Supplementary Table 3). A comparison of model results between corresponding CSF and plasma biomarkers indicated that—in terms of AIC—CSF biomarkers had significantly better-combined prediction of PACC change than plasma biomarkers (AIC_ΔCSF_ = −98 versus AIC_ΔPlasma_ = −28; Supplementary Table 4).

### Modeling clinical progression to AD dementia

**Fig. 2:**
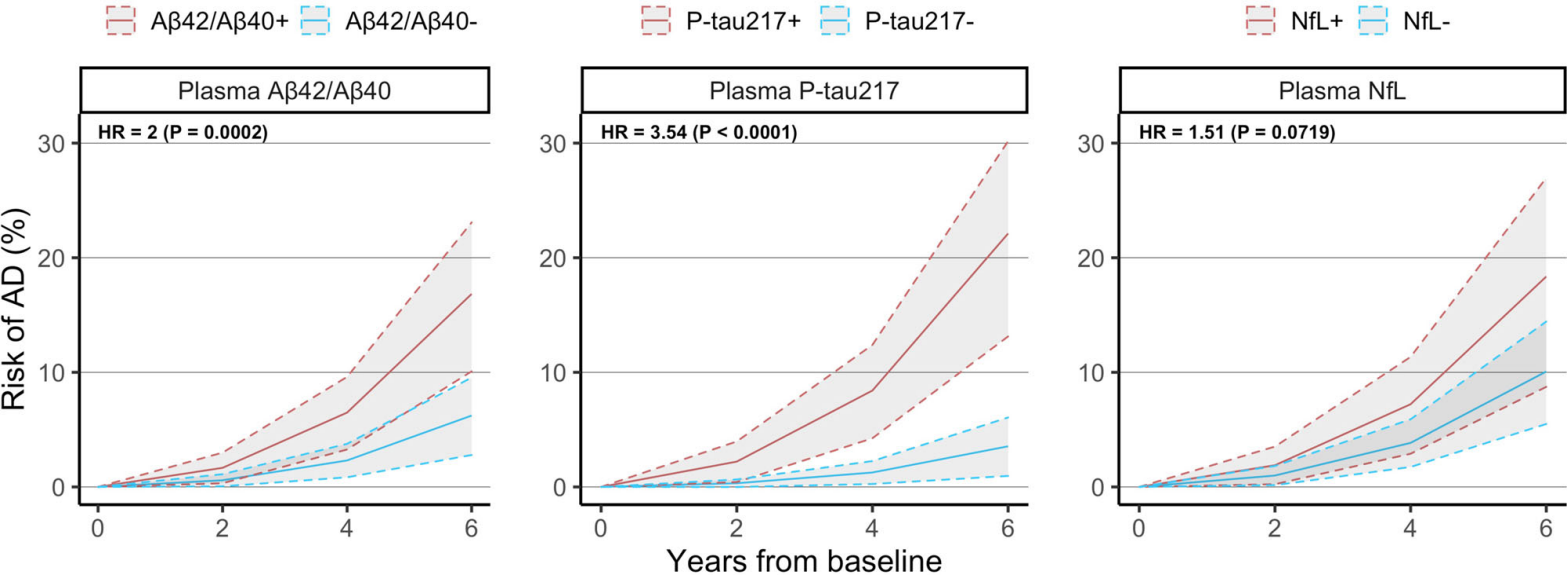
Relationship between plasma biomarkers and conversion to AD. This figure shows the risk of AD dementia overtime expected for a biomarker-negative or biomarker-positive CU individual with average age, average education, and female sex. Hazard ratios at the top left of each panel are presented in terms of “increased risk of converting to AD dementia per standard deviation change in biomarker value” and are derived from Cox regression models with conversion to AD dementia as outcome and age, sex, education, plus each plasma biomarker included separately from each other. All statistical tests were two-sided with no adjustment for multiple comparisons. Shaded areas represent 95% confidence intervals of the regression lines.

We next tested whether plasma biomarkers were significantly associated with subsequent development of AD dementia. When added individually to a basic model consisting of age, sex, and education (Fig. 2), we found that plasma P-tau217 had the strongest association with conversion to AD dementia (HR = 3.54 increased odds/std., *P* < 0.0001), followed by plasma Aβ42/Aβ40 (HR = 2.00, *P* = 0.0002), while plasma NfL on its own did not add significant information to the basic model (HR = 1.51, *P* = 0.07). When combining all three plasma biomarkers together in the same model (Table 3), plasma P-tau217 (HR = 2.97, *P* = 0.0004) and Aβ42/Aβ40 (HR = 1.83, *P* = 0.003) remained significant. When adding *APOE* ε4 status to the set of demographic predictors, AUC values increased for all models, but all plasma biomarkers which were significant in the previous model remained significant when *APOE* ε4 status was added (Supplementary Table 5).

**Table 3:** Association between plasma biomarkers and conversion to AD dementia.

Model	Hazard ratio			AUC [95% CI]	Ref: basic model	
	Plasma Aβ42/Aβ40	Plasma P-tau217	Plasma NfL		*P* value	AIC_Δ_
ATN	1.83 [1.23, 2.74] (*P* = 0.0031)	2.97 [1.62, 5.46] (*P* = 0.0004)	1.07 [0.67, 1.71] (*P* = 0.7898)	0.82 [0.77, 0.91]	<0.0001	−25
A	2.00 [1.39, 2.89] (*P* = 0.0002)			0.77 [0.70, 0.86]	0.0003	−11
T		3.54 [1.98, 6.31] (*P* < 0.0001)		0.77 [0.68, 0.86]	<0.0001	−21
N			1.51 [0.96, 2.35] (*P* = 0.0719)	0.67 [0.60, 0.78]	0.0753	−1

This table shows the results from fitting Cox regression models with conversion to AD as an outcome and plasma biomarkers added separately or all together to a basic model consisting of age, sex, and education. Hazard ratios are presented in terms of “increased risk of converting to AD for each standard deviation change in biomarker value.” AUC values were evaluated and confidence intervals were calculated using 1000 bootstrapped samples. The basic model consisting of only demographics had AUC = 0.64 (95% CI [0.55, 0.77]) and AIC = 274. *P* values represent an ANOVA comparison to the basic model; AIC_Δ_ values represent the change in AIC compared to the basic model and an AIC_Δ_ value of −2 or lower implies a better fit than the basic model. All statistical tests were two-sided with no adjustment for multiple comparisons.

All plasma biomarker-based models significantly improved prediction of 4-year conversion to AD compared to the basic model (AIC = 274; AUC = 0.64 [0.55, 0.77]; see Table 3). In terms of AIC, the model with all three plasma biomarkers together fit the data best (AIC_Δ_ = −25 versus basic model; AUC = 0.82 [0.77, 0.91], *P* < 0.0001), followed by plasma P-tau217 only (AIC_Δ_ = −21; AUC = 0.77 [0.68, 0.86], *P* < 0.0001), plasma Aβ42/Aβ40 only (AIC_Δ_ = −11; AUC = 0.77 [0.70, 0.86], *P* = 0.0003). The model with plasma NfL alone was not significantly better than the basic model (AIC_Δ_ = −1; AUC = 0.67 [0.60, 0.78], *P* = 0.08).

A sensitivity analysis in which conversion to any form of  dementia was used as the outcome of interest produced overall similar results, but with plasma NfL now adding significant prognostic information compared to when predicting conversion to AD dementia (Supplementary Fig. 3; Supplementary Table 6). Another sensitivity analysis in which we additionally adjusted for the effect of SCD on risk of AD dementia resulted in an increased baseline model fit (AUC = 0.86), although this did not affect the statistical significance of any plasma biomarkers or the significant improvement in model fit found by adding plasma biomarkers to the basic model (Supplementary Table 7).

### Power analysis for theoretical clinical trial

**Fig. 3:**
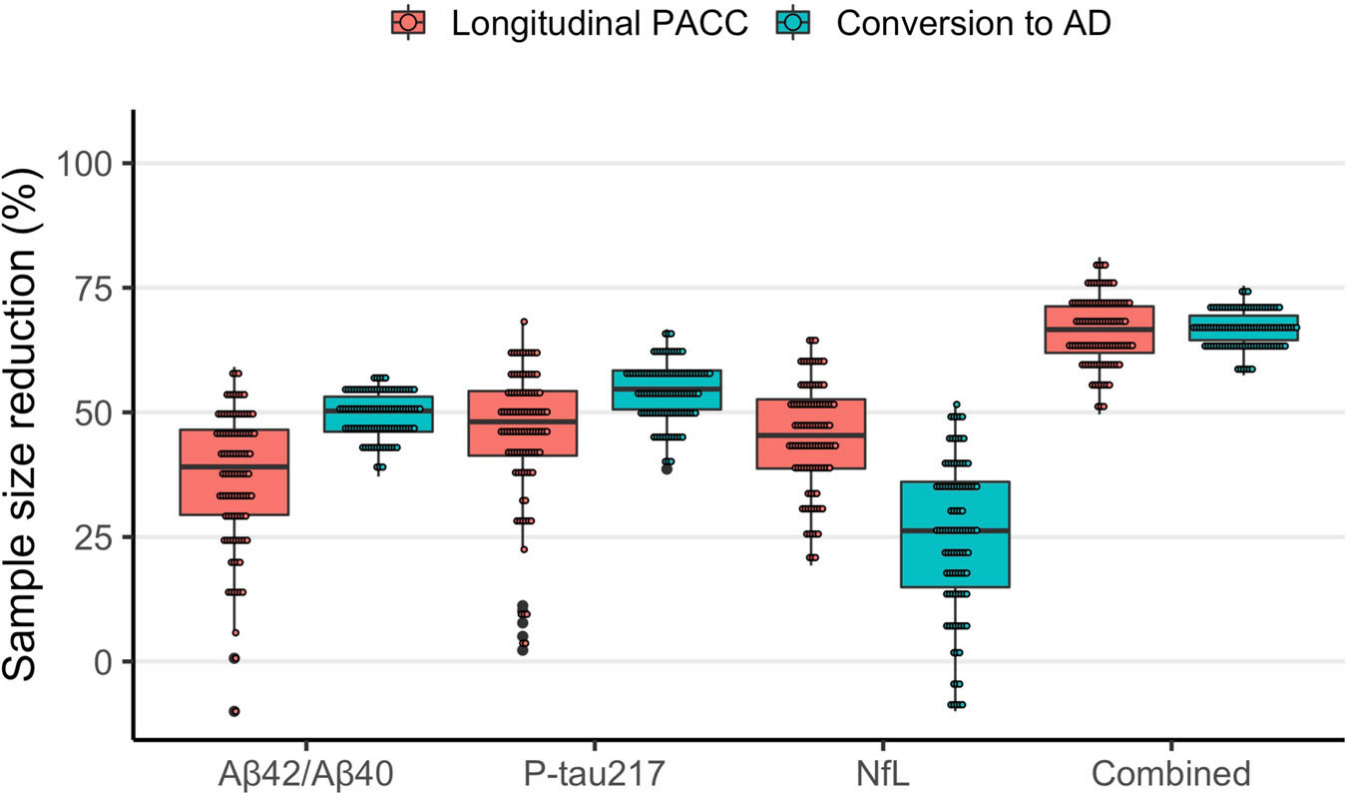
Power enrichment analysis of plasma biomarkers in a theoretical clinical trial. This figure shows the reduction in sample size resulting from using plasma biomarkers for inclusion enrichment in theoretical clinical trials aimed at slowing decline in PACC or reducing risk of AD dementia in a CU population. Sample sizes were estimated for a trial enriched using pre-defined cutoffs for each biomarker as inclusion threshold. Confidence intervals were derived by calculating the 0.025% and 95.75% percentile values from 1000 bootstrapped trials over *n* = 435 individuals. Source data is available in Supplementary Table 8.

**Fig. 4:**
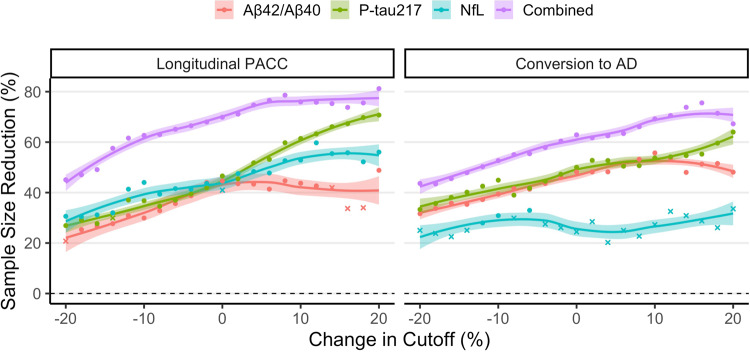
Effect of inclusion threshold variability on biomarker effectiveness. This figure shows the effect that varying the inclusion threshold by 20% in either direction of the pre-defined cutoffs has on the reduction in sample size in a theoretical clinical trial using biomarkers for inclusion enrichment. The reduction in sample size is compared to an unenriched trial with PACC or conversion to AD dementia as primary outcome. For datapoints in the figure, “circles” represent cutoffs for which the required sample size was significantly reduced, while “crosses” represent cutoffs for which required sample size was not significantly reduced. Shaded areas represent 95% confidence intervals of the loess regression lines fit over the entire data range.

Finally, we tested whether using plasma biomarkers for participant screening would reduce the required sample size of a theoretical clinical trial aimed at slowing change in PACC by 30% over 4 years compared to an identical clinical trial without biomarker-based inclusion screening (Fig. 3; Supplementary Table 6). Using pre-defined cutoffs for the biomarker inclusion thresholds, we found that a significantly lower sample size was required to acheive equal power when enriching for plasma P-tau217 (sample size reduction [SS_Δ_] = 47%, CI [16,65], *P* = 0.007), plasma Aβ42/Aβ40 (SS_Δ_ = 45%, CI [20,63], *P* = 0.003) and plasma NfL (SS_Δ_ = 41%, CI [5,63], *P* = 0.03). Moreover, combining all three biomarkers in a multivariable enrichment model led to the largest reduction in sample size (SS_Δ_ = 70% [54,81], *P* < 0.001). All plasma biomarkers were robust to up to 20% change in inclusion threshold and a stricter inclusion threshold led to larger reduction in required sample size (Fig. 4).

For a clinical trial aimed at reducing the risk of conversion to AD by 30% over four years (Fig. 3; Supplementary Table 8), a significant reduction in required sample size was observed when enriching for plasma P-tau217 (SS_Δ_ = 48%, CI [38,56], *P* < 0.001) and plasma Aβ42/Aβ40 (SS_Δ_ = 48%, CI [35,60], *P* = 0.001), while plasma NfL did not significantly enrich such a clinical trial (SS_Δ_ = 24%, CI [−10, 45], *P* = 0.20). An enrichment model which combined all three biomarkers achieved the greatest reduction in sample size (SS_Δ_ = 63%, CI [53,70], *P* < 0.001). Again, all plasma biomarkers besides plasma NfL appeared robust to larger variation in the inclusion threshold (Fig. 4).

Because SCD participants had a significantly higher risk of AD dementia (see Table 1), we also tested whether plasma biomarkers would reduce the required sample size of a clinical trial above and beyond what could be achieved if participants were already screened for SCD status (Supplementary Table 9). To start, screening for SCD status by itself did not lead to a significant reduction in sample size with PACC as trial outcome (SS_Δ_ = 19%, CI [−10, 48], *P* = 0.38), but did lead to a significant reduction in sample size with conversion to AD dementia as trial outcome (SS_Δ_ = 55%, CI [46,62], *P* < 0.001). For PACC, we found an increased effect of plasma Aβ42/Aβ40 (SS_Δ_ = 51%, CI [17,75], *P* = 0.007), plasma NfL (SS_Δ_ = 61%, CI [25,82], *P* = 0.001), and the combined model (SS_Δ_ = 73%, CI [53,87], *P* < 0.001), but a decreased effect of plasma P-tau217 (SS_Δ_ = 44%, CI [−10, 70], *P* = 0.09). With AD dementia as outcome, the plasma Aβ42/Aβ40, plasma P-tau217, and combined models remained significant while the plasma NfL model remained non-significant.

## Discussion

We tested the hypothesis that plasma biomarkers (Aβ42/Aβ40, P-tau217 and NfL) previously established in AD research improve prediction of both cognitive and clinical outcomes for elderly CU individuals. Our primary findings were that plasma biomarkers add significant prognostic information to a basic model consisting only of demographic information (age, sex and education) and can significantly reduce the number of participants required to run AD-related clinical trials in this population.

All three plasma biomarkers were significantly associated with the primary outcomes on their own, but we showed here that they also provide some degree of overlapping information with each other. Plasma P-tau217 and Aβ42/Aβ40 contributed significantly to the prediction of both PACC and risk of AD dementia, while plasma NfL added significant prognostic information for PACC and appeared more useful for predicting general cognitive decline (MMSE) and risk of all-cause dementia. Together, our results demonstrate the wide applicability of this plasma biomarker panel in a CU elderly population.

All three plasma biomarkers also significantly reduced sample sizes needed to run AD clinical trials in a CU population when used for pre-trial inclusion screening. This is a promising result given that clinical trials of AD are increasingly focused on elderly individuals without existing cognitive impairment. Running such a clinical trial in a cost-effective way likely requires the ability to easily and affordably  identify CU individuals at risk of developing AD. Our results suggest that these plasma biomarkers are in fact an effective way to enrich clinical trials in a CU population, and that they even add additional value in a population which has already been enriched for SCD status. While the biomarkers showed promise for stand-alone use for trial enrichment, combining them in a multivariable model led to the greatest reduction in required trial sample size and the lowest variability in estimated sample size reduction.

Interestingly, the combined multivariate model and individual plasma biomarkers were robust to variation in the inclusion threshold, indicating that random shifts in the demographics of a clinical trial screening population or in the analytical variability of the plasma assay may not have a significant negative impact on the effectiveness of such an enrichment scheme. Still, this result should be interpreted with caution since it is not easy to determine how much of the variability is controllable (e.g., through pre-analytical factors) and how much is due to inherent biological variability. It was also not possible to account for the possibility that enrichment may increase study attrition rates or otherwise alter trial dynamics since this type of data is not available to us.

With regards to cognitive outcomes, we found that all three plasma biomarkers added independent information for predicting change in PACC even when combined in the same model. This indicates that if only one or two of the three biomarkers have already been collected in a specific individual, a more accurate prediction can be obtained by additionally measuring the other, unknown biomarker(s). This result was not dependent on the inclusion of *APOE* ε4 status, although including such information did generally raise the prognostic accuracy. Also, plasma Aβ42/Aβ40 had a stronger effect with PACC as outcome rather than MMSE; this might be expected given that PACC was created specifically to pick up early AD-related changes in non-demented individuals^23^ and has been shown to have greater sensitivity to Aβ-related cognitive decline^24^, while the MMSE is a measure of global cognition originally created to differentiate dementia patients from those with psychiatric syndromes^25,26^. This highlights the idea that selection of cognitive scale impacts which plasma biomarkers are most relevant.

With regards to clinical outcomes, we found that both plasma Aβ42/Aβ40 and plasma P-tau217 added independent information for prediction of conversion to AD dementia, even when combined in the same model, further adding to the existing evidence of plasma Aβ42/Aβ40 and P-tau217 as AD-specific markers^27,28^. The contribution of plasma Aβ42/Aβ40 was greatly affected when *APOE* ε4 status was included, but the same effect was not seen for CSF Aβ42/Aβ40, indicating that *APOE* ε4 status may contain prognostic information related to Aβ accumulation which is not picked up by plasma Aβ42/Aβ40^29^. This finding aligns with studies showing a link between *APOE* ε4 and alterations in Aβ-related processes^30,31^. Our results further suggest that *APOE* ε4 status may be informative in a CU population even when plasma biomarker values are available, as prediction of subsequent dementia in particular was significantly better when including *APOE* ε4 as a covariate. The effect of plasma NfL greatly increased when analyzing conversion to all-cause dementia, thereby strengthening previous findings of plasma NfL as a general biomarker of neurodegeneration that detects non-AD-related neurodegenerative changes^32–34^.

We also compared plasma biomarkers directly against their corresponding CSF biomarkers. CSF biomarkers still appear to be superior to plasma biomarkers in terms of cognitive and clinical prognosis in elderly individuals without cognitive impairment, although this comparison was based on Akaike Information Criterion (AIC) values and there was in fact significant overlap in *R*^2^ between models. This indicates that CSF and plasma may provide similar predictive accuracy overall but that CSF provides a much more confident prediction with smaller confidence intervals. We hypothesize that this difference is largely based on the observation that CSF Aβ42/Aβ40 is a much stronger prognostic marker than plasma Aβ42/Aβ40. However, the discrepancy between plasma and CSF Aβ42/Aβ40 may not be biological but may instead be related to the performance of the specific plasma assays used here (Elecsys immunoassay). Our work in MCI patients showed a greater effect of plasma Aβ42/Aβ40 by using a mass-spectrometry method, indicating that a more effective plasma assay could lead to greater value^35^. However, such data were not widely available in this cohort. Further work is needed to compare performance of CSF and plasma biomarkers in CU individuals using different assays, as recent studies suggest that the difference between CSF and plasma biomarkers is less pronounced in MCI ^35^.

Importantly, our results in terms of biomarker effect size did not change when we further took into account the main distinction of cognitively unimpairment—CN versus SCD. To note, both CN and SCD participants are both considered to be CU according to NIA-AA criteria^6^. However, SCD participants in our study were followed in a memory clinic setting and had higher risk of developing AD dementia. Including this diagnostic distinction improved performance of the basic (demographics-only) model but did not alter the finding that plasma biomarkers provide significant prognostic information. Also, enriching for SCD status led to a significant reduction in trial sample size with AD dementia as outcome, but not with cognition as outcome. Since cognition is the main target of current AD trials, this indicates that enriching for SCD alone should not be considered as an adequate replacement for plasma biomarker-based enrichment. In all, our results show that plasma biomarkers are effective in both sub-groups and that it was not simply SCD participants that were driving model performance.

The strengths of this study include the standardized collection and measurement of relevant biomarkers in both plasma and CSF—which allowed for direct comparison between biomarkers and across the two modalities—and the availability of multi-year follow-up data in a large number of participants. Our study improves in particular on previous studies linking plasma P-tau levels on their own with cognitive decline and neurodegeneration in similar populations^22,36^. Understanding the overlapping contributions of biomarkers is therefore a major contribution of our results. Regarding biomarker contributions, however, we note that confidence intervals of individual biomarker effect sizes overlapped greatly within models, indicating that it is not quite possible to determine whether one biomarker was more or less effective in any given model. Therefore, we cannot comment on whether biomarkers should be weighted differently within a prognostic model. Rather, our analysis helps make the decision as to whether a biomarker should be included in such a predictive model at all.

Not having a state-of-the-art mass-spectrometry plasma Aβ42/Aβ40 assay is a weakness, although plasma Aβ42/Aβ40 was still important to the prognostic algorithms. In addition, since evidence of abnormal CSF biomarker levels was required for setting the diagnosis of AD dementia at the memory clinic, it was not possible to fairly compare the performance of plasma and CSF biomarkers in this manner. The evidence we did provide for cognition, however, indicates that CSF biomarkers are still superior to plasma biomarkers in a CU elderly population at least in terms of individual prediction accuracy. Factoring in cost and ease of collection may make plasma more beneficial at a population level. In the future, we hope to validate our findings in an independent cohort as soon as other studies with sufficient longitudinal cognitive follow-up and measurements of plasma P-tau217 become available.

To summarize, our results show that plasma biomarkers relate to AD-related changes in the CU elderly and can significantly reduce sample sizes needed to run clinical trials in such a population. Further work is needed to validate the optimal panel of biomarkers in an eldery CU population, although our preliminary results suggest that all three core plasma biomarkers could be useful.

## Methods

### Participants

The Swedish BioFINDER (Biomarkers for Identifying Neurodegenerative Disorders Early and Reliably; clinical trial no. NCT01208675, www.biofinder.se) cohort used in the present analysis consisted of CN participants with no objective evidence of cognitive impairment at baseline (i.e., healthy controls) and SCD participants who were referred to the memory clinic for investigation but deemed to not have cognitive impairment. The inclusion criteria for CN participants were the following: (i) age ≥65 years; (ii) absence of cognitive symptoms as assessed by a physician with a special interest in cognitive disorders; (iii) MMSE score between 26 and 30 at screening visit; (iv) do not fulfil the criteria for MCI or dementia according to DSM-5 (American Psychiatric Association, 2013); (v) fluent in Swedish. Exclusion criteria include (i) significant unstable systemic illness that makes it difficult to participate in the study; (ii) current significant alcohol or substance misuse; (iii) refusing lumbar puncture or MRI. SCD participants were followed in a memory clinic setting due to cognitive symptoms experienced by the patient and/or informant. These symptoms did not have to be memory complaints, but could also be executive, visuospatial, language, praxis, or psychomotor complaints. After inclusion, patients were categorized as having SCD or MCI based on an extensive neuropsychological battery performed at baseline, examining verbal, episodic memory, visuospatial ability and attention/executive domains. Patients with domain z-score > –1.5 in all domains were classified as SCD and only these participants were included in the present analysis^37^. All data were collected between July 2008 and June 2019.

Conversion to a clinical diagnosis of dementia (AD dementia or dementia due to any cause [“all-cause dementia”]) was evaluated based on physician’s follow-up assessments and reviewed by a consensus group that included memory clinical physicians and a senior neuropsychologist. The diagnosis was made according to the diagnostic and statistical manual of mental disorders version 5 (DSM-5) criteria and required a positive indication of abnormal amyloid accumulation based on CSF Aβ42/Aβ40 levels (cutoff value <0.088 based on mixture modeling statistics)^7^.

All participants had available plasma Aβ42/Aβ40, P-tau217, and NfL measurements and CSF Aβ42/Aβ40, P-tau181, and NfL measurements. All participants gave written informed consent and ethical approval was given by the Regional Ethical Committee in Lund, Sweden.

### Cognitive and clinical outcomes

The primary cognitive outcome was the PACC, as this scale was developed specifically to identify early cognitive changes in individuals without dementia^23^. The PACC score used for the present study was comprised of the MMSE, delayed word recall from the Alzheimer’s Disease Assessment Scale-Cognitive Subscale (weighted double to reflect the emphasis on memory tests in the original PACC), animal fluency, and trail-making B tests and was calculated as described previously^23,38^. The secondary cognitive outcome was the MMSE collected longitudinally during all available follow-up visits. The primary clinical outcome was conversion to AD dementia at any time during longitudinal follow-up. The secondary clinical outcome was conversion to all-cause dementia.

### Plasma and CSF biomarker assays

Biomarkers included in the present study were Aβ42/Aβ40, P-tau217, and NfL measured in plasma, along with Aβ42/Aβ40, P-tau181 (P-tau217 was not available in CSF), and NfL measured in CSF. Plasma Aβ42/Aβ40 was measured using an Elecsys immunoassay on a Cobas e601 analyzer (Roche Diagnostics GmbH, Penzberg, Germany)^7^. Plasma P-tau217 was measured on a Meso-Scale Discovery platform (MSD, Rockville, MD), using an assay developed by Eli Lilly^39^. Plasma NfL was analyzed using a Simoa-based assay^13^. CSF levels of Aβ42/Aβ40 and P-tau181 were measured using Elecsys assays (Roche Diagnostics GmbH), and CSF NfL was measured using the ELISA method (UmanDiagnostics AB, Umeå, Sweden). All biomarker values were treated as continuous measures in all statistical analyses after being transformed using the natural log function and any biomarker values more than four standard deviations away from the mean were excluded.

Biomarker cutoffs were derived from levels at baseline using the Youden index procedure to maximize separation of CU individuals without a positive CSF Aβ42/Aβ40 (*n* = 350) who did not convert to AD dementia versus MCI (*n* = 111) and CU (*n* = 30) individuals who did convert to AD dementia). For plasma Aβ42/Aβ40, the resulting cutoff was 0.066 pg/mL. For Plasma P-tau217, the resulting cutoff was 0.199 pg/mL. For plasma NfL, the resulting cutoff was 22.3 pg/mL. To note, binarized biomarker status was used for visualization purposes only to demonstrate theoretical differences in outcome trajectories.

### Statistical analysis

Linear mixed-effects (LME) modeling was used to model longitudinal PACC, with five models tested in total: a basic model (age, sex, education), the basic model plus each of the three plasma biomarkers added separately (i.e., basic model + Aβ42/Aβ40; basic model + P-tau217; basic model + NfL), and the basic model plus all three plasma biomarkers together. LME models had random intercepts and random slopes with an unstructured covariance matrix. Normality of model residuals was confirmed to ensure appropariateness of LME modeling. Cox regression was used to model clinical conversion to AD dementia with the same models fit as above; all participants were right censored at last follow-up visit or at clinical conversion. A power analysis was performed in which the reduction in sample size needed to achieve 80% power to observe a 30% reduction in cognitive decline or clinical conversion was calculated in a scenario where each biomarker was used individually (based on univariate thresholds) or combined (based on a fitted multivariate model) to screen individuals for trial inclusion and in order to enrich the trial population for the relevant outcome. All biomarkers were treated as continuous variables after being log-transformed prior to any analysis.

The primary cognitive outcome was also analyzed using CSF biomarkers on the same subjects and results were compared between CSF and plasma models using the AIC. The larger the difference in AIC is between two models, the less plausible it is that the model with higher AIC provides the best fit^40–42^. The risk of AD dementia was not analyzed using CSF biomarkers because these biomarkers were consulted by neurologists during diagnosis, leading to potential circular reasoning.

Sensitivity analyses were also performed with (a) MMSE as the cognitive outcome instead of PACC, (b) all-cause dementia as the clinical outcome instead of AD dementia, and (c) *APOE* ε4 status (one or more ε4 copy versus zero ε4 copies) included as an additional predictor in the basic model.

All analyses were performed using the *ABA* (“Automated Biomarker Analysis”) package (v1.0.1) written in the R programming language (v4.0.0). All statistical tests were two-sided with a significance level of 0.05.

### Reporting summary

Further information on research design is available in the Nature Research Reporting Summary linked to this article.

## Supplementary information

Supplementary Information

Reporting Summary

## Data Availability

All relevant source data from the present manuscript along with anonymized data from the BioFINDER study will be shared by request from a qualified academic investigator for the sole purpose of replicating procedures and results presented in the article and as long as data transfer is in agreement with EU legislation on the general data protection regulation and decisions by the Ethical Review Board of Sweden and Region Skåne, which should be regulated in a material transfer agreement. The code used for statistical analyses is available at a public repository.

## Code availability

Partial code used for statistical analyses is available at a public repository (www.github.com/ncullen93/ATNCU). Running the full code requires access to the source data, which is available upon request from qualified academic investigators. This code is released without warranty and without requirement for citation or inclusion.
