# Supplementary material for: Plasma biomarkers of Alzheimer’s disease improve prediction of cognitive decline in cognitively unimpaired elderly populations
Source: Nat Commun. 2021 Jun 11;12:3555. doi: 10.1038/s41467-021-23746-0 (PMC8196018; doi:10.1038/s41467-021-23746-0)
Supplement: Supplementary file 1 — Supplementary Information [file 41467_2021_23746_MOESM1_ESM.pdf]

Supplementary Materials

Plasma biomarkers of Alzheimer's disease improve prediction of cognitive decline in cognitively unimpaired elderly populations

Nicholas C. Cullen, B.S.[1], Antoine Leuzy, Ph.D.[1], Shorena Janelidze, Ph.D.[1], Sebastian Palmqvist, M.D.[1,2], Anna L. Svenningsson, M.D.[1,2], Erik Stomrud, M.D.[1,2], Jeffrey L. Dage, Ph.D.[3], Niklas Mattsson-Carlgren, M.D.[1,4,5, †], Oskar Hansson, M.D.[1,2, †]

[1]Lund University, Clinical Memory Research Unit, Lund, Sweden; [2]Memory Clinic, Skåne University Hospital, Lund, Sweden; [3]Eli Lilly and Company, Indianapolis, IN, USA; [4]Department of Neurology, Skåne University Hospital, Lund, Sweden; [5]Wallenberg Centre for Molecular Medicine, Lund University, Lund, Sweden

[†] Contributed equally as senior authors

Supplementary Tables

Supplementary Table 1. Association between plasma biomarkers and longitudinal PACC with additional covariate adjustment for APOE e4 status

Model	Beta Coefficient			R^2 [95% CI]	Ref: Basic Model	
	Plasma Aβ42/Aβ40	Plasma P-tau217	Plasma NfL		P value	AIC∆
ATN	-0.15 [0.05, 0.24] (P=0.0025)	-0.15 [-0.25, -0.06] (P=0.0020)	-0.12 [-0.21, -0.02] (P=0.0142)	0.15 [0.12, 0.17]	<0.0001	-28
A	-0.19 [0.09, 0.28] (P=0.0001)			0.11 [0.09, 0.14]	0.0002	-14
T		-0.20 [-0.30, -0.11] (P<0.0001)		0.10 [0.08, 0.13]	0.0001	-14
N			-0.15 [-0.25, -0.06] (P=0.0017)	0.10 [0.09, 0.14]	0.0016	-9

This table shows the results from fitting linear mixed effects models with longitudinal PACC as outcome and plasma biomarkers added separately or all together to a basic model consisting of age, sex, education, and *APOE* status. Beta coefficients are presented in terms of “PACC points / year per standard deviation change in biomarker value.” R^2 values were evaluated at the four-year follow-up point, and confidence intervals were calculated using 1000 bootstrapped samples. The basic model consisting of only demographics had R^2 = 0.07 (95% CI [0.07, 0.11]) and AIC = 6700. Legend: P-values represent an ANOVA comparison to the basic model; AIC∆ values represent the change in AIC compared to the basic model and an AIC∆ value of -2 or lower implies a better fit than the basic model. All statistical tests were two-sided with no adjustment for multiple comparisons.

Supplementary Table 2. Association between plasma biomarkers and longitudinal MMSE

Model	Beta Coefficient			R^2 [95% CI]	Ref: Basic Model	
	**Plasma Aβ42/Aβ40**	**Plasma P-tau217**	**Plasma NfL**		**P value**	**AIC_{Δ}**
ATN	-0.05 [-0.10, -0.01] (P=0.0238)	-0.06 [-0.11, -0.01] (P=0.0122)	-0.07 [-0.12, -0.03] (P=0.0026)	0.10 [0.06, 0.11]	<0.0001	-21
A	-0.07 [-0.12, -0.03] (P=0.0022)			0.06 [0.04, 0.07]	0.0015	-10
T		-0.09 [-0.13, -0.04] (P=0.0004)		0.07 [0.03, 0.08]	0.001	-10
N			-0.09 [-0.14, -0.04] (P=0.0003)	0.06 [0.03, 0.09]	0.001	-10

This table shows the results from fitting linear mixed effects models with longitudinal MMSE as outcome and plasma biomarkers added separately or all together to a basic model consisting of age, sex, and education. Beta coefficients are presented in terms of "MMSE points / year per standard deviation change in biomarker value." R^2 values were evaluated at the four-year follow-up point, and confidence intervals were calculated using 1000 bootstrapped samples. The basic model consisting of only demographics had R^2 = 0.04 (95% CI [0.02, 0.05]) and AIC = 4702. Legend: P-values represent an ANOVA comparison to the basic model; AIC_{Δ} values represent the change in AIC compared to the basic model and an AIC_{Δ} value of -2 or lower implies a better fit than the basic model. All statistical tests were two-sided with no adjustment for multiple comparisons.

Supplementary Table 3. Association between plasma biomarkers and longitudinal PACC with additional covariate adjustment for diagnostic status

Model	Beta Coefficient			R^2 [95% CI]	Ref: Basic Model	
	Plasma Aβ42/Aβ40	Plasma P-tau217	Plasma NfL		P value	AIC∆
ATN	-0.14 [-0.24, -0.05] (P=0.0029)	-0.16 [-0.25, -0.06] (P=0.0011)	-0.13 [-0.23, -0.04] (P=0.0056)	0.22 [0.18, 0.26]	<0.0001	-30
A	-0.19 [-0.28, -0.09] (P=0.0001)			0.20 [0.16, 0.24]	0.0001	-14
T		-0.21 [-0.30, -0.12] (P<0.0001)		0.18 [0.15, 0.22]	0.0001	-15
N			-0.17 [-0.26, -0.07] (P=0.0005)	0.19 [0.15, 0.23]	0.0006	-11

This table shows the results from fitting linear mixed effects models with longitudinal PACC as outcome and plasma biomarkers added separately or all together to a basic model consisting of age, sex, and education, along with additional adjustment for diagnostic status on both baseline and change in PACC. Beta coefficients are presented in terms of "PACC points / year per standard deviation change in biomarker value." R^2 values were evaluated at the four-year follow-up point, and confidence intervals were calculated using 1000 bootstrapped samples. The basic model consisting of only demographics had R^2 = 0.17 (95% CI [0.13, 0.21]) and AIC = 6644. Legend: P-values represent an ANOVA comparison to the basic model; AIC∆ values represent the change in AIC compared to the basic model and an AIC∆ value of -2 or lower implies a better fit than the basic model. All statistical tests were two-sided with no adjustment for multiple comparisons.

Supplementary Table 4. Association between CSF biomarkers and longitudinal PACC

Model	Beta Coefficient			R^2 [95% CI]	Ref: Basic Model	
	Plasma Aβ42/Aβ40	Plasma P-tau217	Plasma NfL		P value	AIC_Δ
ATN	-0.26 [-0.34, -0.17] (P<0.0001)	-0.20 [-0.30, -0.10] (P=0.0001)	-0.20 [-0.30, -0.11] (P<0.0001)	0.25 [0.21, 0.28]	<0.0001	-98
A	-0.25 [-0.34, -0.16] (P<0.0001)			0.13 [0.11, 0.17]	<0.0001	-30
T		-0.28 [-0.37, -0.18] (P<0.0001)		0.14 [0.12, 0.17]	<0.0001	-34
N			-0.30 [-0.39, -0.21] (P<0.0001)	0.18 [0.14, 0.21]	<0.0001	-52

This table shows the results from fitting linear mixed effects models with longitudinal PACC as outcome and CSF biomarkers added separately or all together to a basic model consisting of age, sex, and education. Beta coefficients are presented in terms of "PACC points / year per standard deviation change in biomarker value." R^2 values were evaluated at the four-year follow-up point, and confidence intervals were calculated using 1000 bootstrapped samples. The basic model consisting of only demographics had $R^2 = 0.07$ (95% CI [0.06, 0.11]) and AIC = 6699. Legend: P-values represent an ANOVA comparison to the basic model; AIC_Δ values represent the change in AIC compared to the basic model and an AIC_Δ value of -2 or lower implies a better fit than the basic model. All statistical tests were two-sided with no adjustment for multiple comparisons.

Supplementary Table 5. Association between plasma biomarkers and conversion to AD dementia with additional covariate adjustment for APOE e4 status

**Model**	**Hazard Ratio**			**AUC [95% CI]**	**Ref: Basic Model**	
	**Plasma Aβ42/Aβ40**	**Plasma P-tau217**	**Plasma NfL**		**P value**	**AIC$_{\Delta}$**
ATN	1.57 [1.02, 2.43] (P=0.0423)	2.71 [1.44, 5.10] (P=0.0021)	1.09 [0.67, 1.78] (P=0.7202)	0.86 [0.82, 0.93]	0.0002	-14
A	1.69 [1.13, 2.55] (P=0.0114)			0.81 [0.77, 0.89]	0.0141	-4
T		2.99 [1.64, 5.48] (P=0.0004)		0.84 [0.75, 0.91]	0.0001	-13
N			1.47 [0.95, 2.29] (P=0.0859)	0.79 [0.72, 0.89]	0.0918	-1

This table shows the results from fitting Cox regression models with conversion to AD as outcome and plasma biomarkers added separately or all together to a basic model consisting of age, sex, education, and *APOE* status. Hazard ratios are presented in terms of "increased risk of converting to AD for each standard deviation change in biomarker value." AUC values were evaluated at the four-year follow-up point, and confidence intervals were calculated using 1000 bootstrapped samples. The basic model consisting of only demographics had AUC = 0.78 (95% CI [0.70, 0.91]) and AIC = 253. Legend: P-values represent an ANOVA comparison to the basic model; AIC$_{\Delta}$ values represent the change in AIC compared to the basic model and an AIC$_{\Delta}$ value of -2 or lower implies a better fit than the basic model. All statistical tests were two-sided with no adjustment for multiple comparisons.

Supplementary Table 6. Association between plasma biomarkers and conversion to all-cause dementia

Model	Hazard Ratio			AUC [95% CI]	Ref: Basic Model	
	Plasma Aβ42/Aβ40	Plasma P-tau217	Plasma NfL		P value	AIC_{Δ}
ATN	1.72 [1.22, 2.42] (P=0.0019)	1.93 [1.21, 3.07] (P=0.0055)	1.25 [0.85, 1.85] (P=0.2617)	0.75 [0.68, 0.84]	<0.0001	-21
A	1.84 [1.34, 2.53] (P=0.0002)			0.73 [0.67, 0.82]	0.0002	-11
T		2.36 [1.50, 3.71] (P=0.0002)		0.72 [0.63, 0.82]	<0.0001	-14
N			1.54 [1.06, 2.24] (P=0.0246)	0.68 [0.60, 0.78]	0.0274	-3

This table shows the results from fitting Cox regression models with conversion to all-cause dementia as outcome and plasma biomarkers added separately or all together to a basic model consisting of age, sex, and education. Hazard ratios are presented in terms of "increased risk of converting to all-cause dementia for each standard deviation change in biomarker value." AUC values were evaluated at the four-year follow-up point, and confidence intervals were calculated using 1000 bootstrapped samples. The basic model consisting of only demographics had AUC = 0.66 (95% CI [0.57, 0.75]) and AIC = 368. Legend: P-values represent an ANOVA comparison to the basic model; AIC_{Δ} values represent the change in AIC compared to the basic model and an AIC_{Δ} value of -2 or lower implies a better fit than the basic model. All statistical tests were two-sided with no adjustment for multiple comparisons.

Supplementary Table 7. Association between plasma biomarkers and conversion to AD dementia with additional covariate adjustment for diagnostic status

Model	Hazard Ratio			AUC [95% CI]	Ref: Basic Model	
	Plasma Aβ42/Aβ40	Plasma P-tau217	Plasma NfL		P value	AIC_{Δ}
ATN	1.76 [1.13, 2.75] (P=0.0119)	3.27 [1.75, 6.11] (P=0.0002)	1.12 [0.64, 1.97] (P=0.6808)	0.92 [0.89, 0.95]	<0.0001	-27
A	1.90 [1.28, 2.82] (P=0.0014)			0.89 [0.85, 0.93]	0.0015	-8
T		3.83 [2.10, 6.97] (P<0.0001)		0.91 [0.87, 0.95]	<0.0001	-24
N			1.60 [1.00, 2.55] (P=0.0493)	0.86 [0.81, 0.91]	0.0558	-2

This table shows the results from fitting Cox regression models with conversion to AD as outcome and plasma biomarkers added separately or all together to a basic model consisting of age, sex, education, and *APOE* status, along with additional adjustment for diagnostic status. Hazard ratios are presented in terms of "increased risk of converting to AD for each standard deviation change in biomarker value." AUC values were evaluated at the four-year follow-up point, and confidence intervals were calculated using 1000 bootstrapped samples. The basic model consisting of only demographics had AUC = 0.86 (95% CI [0.80, 0.92]) and AIC = 243. Legend: P-values represent an ANOVA comparison to the basic model; AIC_{Δ} values represent the change in AIC compared to the basic model and an AIC_{Δ} value of -2 or lower implies a better fit than the basic model. All statistical tests were two-sided with no adjustment for multiple comparisons.

Supplementary Table 8. Power analysis for a theoretical clinical trial

**Trial Endpoint**	**Biomarker**	**Sample Size Reduction (%)**	**P-value**
Change in PACC	Plasma Aβ42/Aβ40	45 [20, 63]	0.0032
	Plasma P-tau217	47 [16, 65]	0.0072
	Plasma NfL	41 [5, 63]	0.028
	Combined Model	70 [54, 81]	< 0.001
Conversion to AD	Plasma Aβ42/Aβ40	48 [38, 56]	< 0.001
	Plasma P-tau217	50 [35, 60]	0.0008
	Plasma NfL	24 [-10, 45]	0.2096
	Combined Model	63 [53, 70]	< 0.001

This tables shows the reduction in sample size resulting from using plasma biomarkers for inclusion enrichment in theoretical clinical trials aimed at slowing decline in PACC or reducing risk of conversion to AD dementia in a CU population. Sample sizes were estimated for a clinical trial in which pre-defined cutoffs for each biomarker were used as a screening inclusion threshold. Sample size reductions presented in the table are for the enriched trial relative to a trial which does not use any biomarkers for screening/enrichment. Pre-defined threshold values are described in the methods section. Confidence intervals were derived using 1000 bootstrapped trials. All statistical tests were two-sided with no adjustment for multiple comparisons.

Supplementary Table 9. Power analysis for a theoretical clinical trial in SCD individuals only

Trial Endpoint	Biomarker	Sample Size Reduction (%)	P-value
Change in PACC	Plasma Aβ42/Aβ40	51 [17, 75]	0.0072
	Plasma P-tau217	44 [-10, 70]	0.0976
	Plasma NfL	61 [25, 82]	0.0096
	Combined Model	73 [53, 87]	< 0.001
Conversion to AD	Plasma Aβ42/Aβ40	48 [37, 58]	< 0.001
	Plasma P-tau217	45 [25, 58]	0.004
	Plasma NfL	29 [-10, 50]	0.1528
	Combined Model	61 [50, 70]	< 0.001

This tables shows the reduction in sample size resulting from using plasma biomarkers for inclusion enrichment in theoretical clinical trials aimed at slowing decline in PACC or reducing risk of conversion to AD dementia in a CU population which has already been screened for subjective cognitive decline (SCD). Sample sizes were estimated for a clinical trial in which pre-defined cutoffs for each biomarker were used as a screening inclusion threshold. Sample size reductions presented in the table are for the enriched trial relative to a trial which does not use any biomarkers for screening/enrichment. Pre-defined threshold values are described in the methods section. Confidence intervals were derived using 1000 bootstrapped trials. All statistical tests were two-sided with no adjustment for multiple comparisons.

Supplementary Figures

Supplementary Figure 1. Relationship between plasma biomarkers

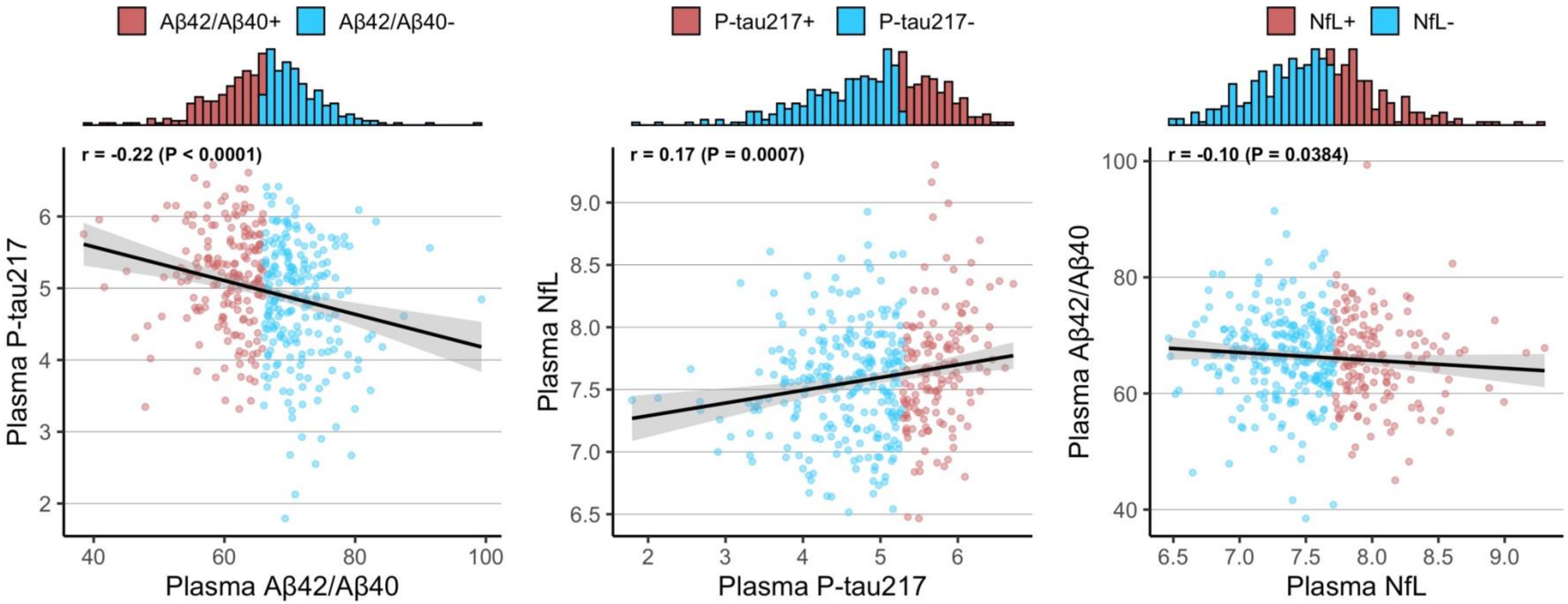

This figure shows the relationship between each pair of plasma biomarkers in the study population. All biomarkers were natural log-transformed and statistical associations were tested using Spearman correlation. The upper panels show the histogram distributions for each corresponding biomarker labelled on the x-axis and each individual is colored based on biomarker status (positive or negative; defined using pre-defined

cutoffs) for the biomarker on the x-axis. All statistical tests were two-sided with no adjustment for multiple comparisons. Shaded areas represent 95% confidence intervals of the regression lines.

Supplementary Figure 2. Relationship between plasma biomarkers and change in MMSE

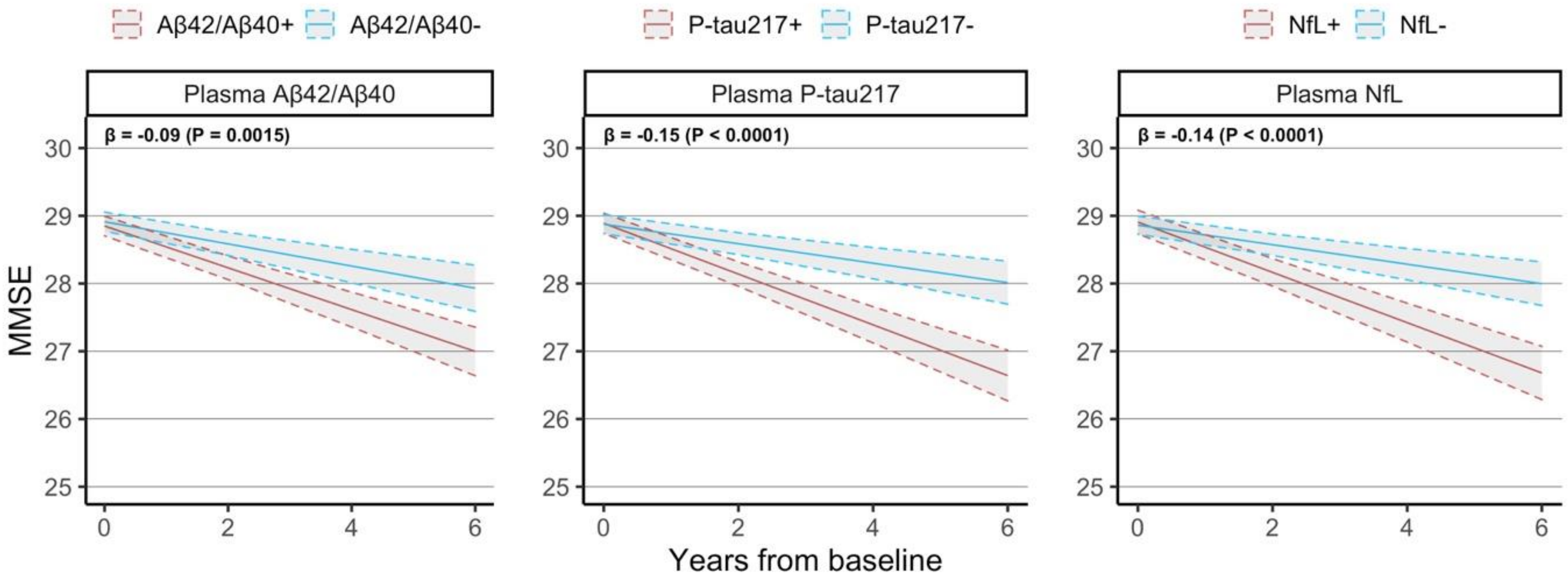

This figure shows the longitudinal MMSE trajectory estimated for a CU individual with average age, average education, female sex and either biomarker-negative or biomarker-positive. Beta coefficients at the top left of each panel are presented in terms of “points / year per standard deviation change in biomarker value” and are derived from linear mixed effects models with longitudinal MMSE as outcome and age, sex,

education, plus each plasma biomarker included separately from each other. All statistical tests were two-sided with no adjustment for multiple comparisons. Shaded areas represent 95% confidence intervals of the regression lines.

Supplementary Figure 3. Relationship between plasma biomarkers and conversion to all-cause dementia

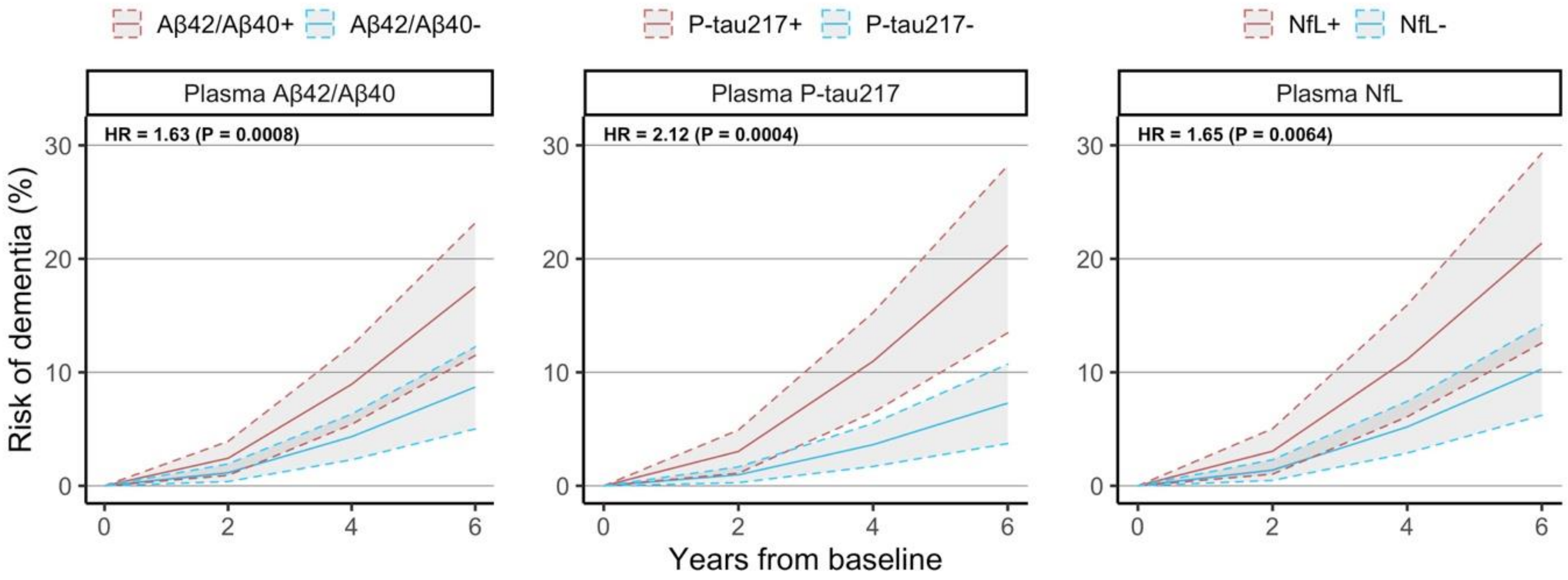

This figure shows the conversion to all-cause dementia estimated for a CU individual with average age, average education, female sex and either biomarker-negative or biomarker-positive. Hazard ratios at the top left of each panel are presented in terms of "increased risk of converting to all-cause dementia per standard deviation change in biomarker value" and are derived from Cox regression models with conversion to all-cause dementia as outcome and age, sex, education, plus each plasma biomarker included separately from each other. All statistical tests were two-sided with no adjustment for multiple comparisons. Shaded areas represent 95% confidence intervals of the regression lines.
