# Supplementary material for: Plasma biomarkers of Alzheimer’s disease improve prediction of cognitive decline in cognitively unimpaired elderly populations
Source: Nat Commun. 2021 Jun 11;12:3555. doi: 10.1038/s41467-021-23746-0 (PMC8196018; doi:10.1038/s41467-021-23746-0)
Supplement: Supplementary file 2 — Reporting Summary [file 41467_2021_23746_MOESM2_ESM.pdf]

nature research

Corresponding author(s): Niklas Matsson-Carlgren, Oskar Hansson

Last updated by author(s): May 3, 2021

Reporting Summary

Nature Research wishes to improve the reproducibility of the work that we publish. This form provides structure for consistency and transparency in reporting. For further information on Nature Research policies, see our Editorial Policies and the Editorial Policy Checklist.

Statistics

For all statistical analyses, confirm that the following items are present in the figure legend, table legend, main text, or Methods section.

n/a	Confirmed	
☐	☒	The exact sample size (*n*) for each experimental group/condition, given as a discrete number and unit of measurement
☐	☒	A statement on whether measurements were taken from distinct samples or whether the same sample was measured repeatedly
☐	☒	The statistical test(s) used AND whether they are one- or two-sided<br>*Only common tests should be described solely by name; describe more complex techniques in the Methods section.*
☐	☒	A description of all covariates tested
☐	☒	A description of any assumptions or corrections, such as tests of normality and adjustment for multiple comparisons
☐	☒	A full description of the statistical parameters including central tendency (e.g. means) or other basic estimates (e.g. regression coefficient) AND variation (e.g. standard deviation) or associated estimates of uncertainty (e.g. confidence intervals)
☐	☒	For null hypothesis testing, the test statistic (e.g. *F*, *t*, *r*) with confidence intervals, effect sizes, degrees of freedom and *P* value noted<br>*Give P values as exact values whenever suitable.*
☒	☐	For Bayesian analysis, information on the choice of priors and Markov chain Monte Carlo settings
☒	☐	For hierarchical and complex designs, identification of the appropriate level for tests and full reporting of outcomes
☐	☒	Estimates of effect sizes (e.g. Cohen's *d*, Pearson's *r*), indicating how they were calculated

Our web collection on statistics for biologists contains articles on many of the points above.

Software and code

Policy information about availability of computer code

Data collection	Data is exported to csv-files and can then be received from the BioFINDER team upon request.
Data analysis	All analyses were performed using the R programming language (v4.0.0) and the Automated Biomarker Analysis (ABA) package (v1.0.1). The code used for statistical analyses will be made available at a public repository (github.com/ncullen93/ATNCU) upon publication.

For manuscripts utilizing custom algorithms or software that are central to the research but not yet described in published literature, software must be made available to editors and reviewers. We strongly encourage code deposition in a community repository (e.g. GitHub). See the Nature Research guidelines for submitting code & software for further information.

Data

Policy information about availability of data

All manuscripts must include a data availability statement. This statement should provide the following information, where applicable:

- Accession codes, unique identifiers, or web links for publicly available datasets
- A list of figures that have associated raw data
- A description of any restrictions on data availability

Anonymized data from the BioFINDER study will be shared by request from a qualified academic investigator for the sole purpose of replicating procedures and results presented in the article and as long as data transfer is in agreement with EU legislation on the general data protection regulation and decisions by the Ethical Review Board of Sweden and Region Skåne, which should be regulated in a material transfer agreement. The code used for statistical analyses is available at a public repository.

Field-specific reporting

Please select the one below that is the best fit for your research. If you are not sure, read the appropriate sections before making your selection.

- [x] Life sciences
- [ ] Behavioural & social sciences
- [ ] Ecological, evolutionary & environmental sciences

For a reference copy of the document with all sections, see nature.com/documents/nr-reporting-summary-flat.pdf

Life sciences study design

All studies must disclose on these points even when the disclosure is negative.

Sample size	We included all subjects who had data available for the variables of interest in BioFINDER.
Data exclusions	Individuals in the cohort without available plasma measurements were excluded from the present analysis. All exclusion criteria established prior to performing any statistical analysis.
Replication	Validation was done using bootstrapping sampling during initial submission and once more during the manuscript revision. The analysis has not been replicated in other cohorts due to the lack of availability of all plasma assays.
Randomization	The cohort used in the present analysis consisted of CU individuals (i.e. no objective evidence of cognitive impairment at baseline) and SCD individuals (i.e. individuals who were referred to the memory clinic for investigation but deemed to not have cognitive impairment) recruited consecutively. Exclusion criteria included cognitive impairment. After their baseline visit, all patients were seen annually in order to assess clinical progression. Demographic covariates such as age, sex, and education were controlled for in all statistical analysis.
Blinding	CSF and plasma analyses were performed by technicians blinded to clinical data. Blinding during initial collection of fluid biomarker samples along with cognitive measurements was not possible due to the fact that impaired individuals were seen in a memory clinic setting.

Reporting for specific materials, systems and methods

We require information from authors about some types of materials, experimental systems and methods used in many studies. Here, indicate whether each material, system or method listed is relevant to your study. If you are not sure if a list item applies to your research, read the appropriate section before selecting a response.

Materials & experimental systems

n/a	Involved in the study	
[ ]	[x]	Antibodies
[x]	[ ]	Eukaryotic cell lines
[x]	[ ]	Palaeontology and archaeology
[x]	[ ]	Animals and other organisms
[ ]	[x]	Human research participants
[ ]	[x]	Clinical data
[x]	[ ]	Dual use research of concern

Methods

n/a	Involved in the study	
[x]	[ ]	ChIP-seq
[x]	[ ]	Flow cytometry
[x]	[ ]	MRI-based neuroimaging

Antibodies

Antibodies used	Complete details for the assays used in the analysis of plasma and CSF can be found in the following publications: for BioFinder, plasma Aβ42/Aβ40 (Palmqvist et al., JAMA Neurol 2019), plasma P-tau217 (Palmqvist et al., JAMA 2020), plasma NfL (Mattsson et al., JAMA Neurol 2019), CSF Aβ42 and P-tau181 (Hansson et al. Alzheimers Dement 2018), CSF NfL (Mattsson et al., EMBO Mol Med 2016).
Validation	Validation details for the assays used in the analysis of plasma and CSF can be found in the following publications: for BioFinder, plasma Aβ42/Aβ40 (Palmqvist et al., JAMA Neurol 2019), plasma P-tau217 (Palmqvist et al., JAMA 2020), plasma NfL (Mattsson et al., JAMA Neurol 2019), CSF Aβ42 and P-tau181 (Hansson et al. Alzheimers Dement 2018), CSF NfL (Mattsson et al., EMBO Mol Med 2016).

Human research participants

Policy information about studies involving human research participants

Population characteristics	A total of 435 CU individuals were included of which 167 (38.4%) had subjective cognitive decline (SCD). During follow-up, a total of 28 individuals converted to AD dementia (6.4%) and 39 individuals converted to all-cause dementia (9.0%). Participant characteristics are provided in Table 1.
Recruitment	MCI patients from BioFINDER were recruited and evaluated at the memory clinics in the cities of Lund, Malmö and Ängelholm between July 2008 and June 2019. Self-selection bias could be present because CU individuals who participate in such a clinical study as controls may not represent the general population completely. However, the large sample size available likely mitigates the effects of potential bias as does the fact that fluid biomarkers are objective measures of pathology.
Ethics oversight	For BioFINDER, ethical approval was given by the Regional Ethical Committee of Lund University.

Note that full information on the approval of the study protocol must also be provided in the manuscript.

Clinical data

Policy information about clinical studies

All manuscripts should comply with the ICMJEguidelines for publication of clinical research and a completedCONSORT checklist must be included with all submissions.

Clinical trial registration	NCT01208675 (BioFINDER)
Study protocol	Clinical protocols can be obtained online: BioFINDER, www.biofinder.se
Data collection	For BioFINDER, all data was collected between July 2008 and June 2019. These dates include clinical evaluations. Full details are provided at https://clinicaltrials.gov/ct2/show/NCT01208675?term=biofinder&rank=2 (BioFINDER).
Outcomes	The primary cognitive outcome was Pre-Alzheimer Clinical Composite (PACC) and the secondary cognitive outcome was the Mini-Mental State Examination (MMSE). The primary clinical outcome was development of Alzheimer's disease dementia and the secondary clinical outcome was development of any type of dementia. Full details are provided at https://clinicaltrials.gov/ct2/show/NCT01208675?term=biofinder&rank=2 (BioFINDER).
